# 

**Published:** 1806-10

**Authors:** 


### NEW MEDICAL PUBLICATIONS.

The Naval Military and private Practitioner's Amanuensis Medicus et Chirurgicus, by Dr. Cuming, 7s. boards.

The Philadelphia Medical Museum, conducted by John Redman Coxe, M. D. Vol. 1 and 2, 10s. 6d. boards.

---

### *TO CORRESPONDENTS.*

Mr. Merriman's Cases of Retroversion of the Womb; Observations on Dr. Stone's Treatise on Diseases of the Stomach; Mr. Young's Reply to Dr. Adams; and Observations on Medical Reform, by a Practitioner;—will appear in our next Number.

Communications are also received from Mr. Chalmers, J. W. O. Mr. Denmark, Mr. C. Thomas, Mr. Smart, Mr. C. M. Clarke, Dr. Dancer in Reply to Dr. Grant, and Aminicus on Ching's lozenges.

